# Recognition of Dimeric Lewis X by Anti-Dimeric Le^x^ Antibody SH2

**DOI:** 10.3390/vaccines8030538

**Published:** 2020-09-17

**Authors:** Sinthuja Jegatheeswaran, Ari Asnani, Adam Forman, Jenifer L. Hendel, Christopher J. Moore, Ali Nejatie, An Wang, Jo-Wen Wang, France-Isabelle Auzanneau

**Affiliations:** 1 Department of Chemistry, University of Guelph, Guelph, ON N1G 2W1, Canada; sinthuja.jegatheeswaran@mail.utoronto.ca (S.J.); ari.asnani@unsoed.ac.id (A.A.); adam.forman@mail.utoronto.ca (A.F.); jeniferhendel@gmail.com (J.L.H.); chris.moore.9912@gmail.com (C.J.M.); alinejatie@gmail.com (A.N.); eric.wang@sgs.com (A.W.); wangjowen@gmail.com (J.-W.W.); 2 Immunology Department, University of Toronto, 1 King’s College Circle, Toronto, ON M5S-1A8, Canada; 3 Department of Chemistry, Universitas Jenderal Soedirman, Purwokerto, Jawa Tengah 53123, Indonesia; 4 Department of Chemistry, University of Toronto, 80 St. George Street, Toronto, ON M5S-3H6, Canada; 5 Research and Development, Ludger Ltd., Culham Science Centre, Abingdon, Oxfordshire OX14-3EB, UK; 6 Quality Control, SteriMax Inc., 2770 Portland Dr, Oakville, ON L6H-6R4, Canada; 7 Department of Chemistry, Simon Fraser University, Burnaby, BC V5A1S6, Canada; 8 SGS-CSTC Standards Technical Services Co., Ltd. 4/F, 4th Building, 889 Yishan Road, Xuhui District, Shanghai 200233, China; 9 IQVIA, QuintilesIMS, Clinical Research, 10188 Telesis Ct #400, San Diego, CA 92121, USA

**Keywords:** tumor-associated carbohydrate antigens, ELISA, dimeric Lewis X, SH2, conformational epitope

## Abstract

The carbohydrate antigen dimeric Lewis X (DimLe^x^), which accumulates in colonic and liver adenocarcinomas, is a valuable target to develop anti-cancer therapeutics. Using the native DimLe^x^ antigen as a vaccine would elicit an autoimmune response against the Le^x^ antigen found on normal, healthy cells. Thus, we aim to study the immunogenic potential of DimLe^x^ and search internal epitopes displayed by DimLe^x^ that remain to be recognized by anti-DimLe^x^ monoclonal antibodies (mAbs) but no longer possess epitopes recognized by anti-Le^x^ mAbs. In this context, we attempted to map the epitope recognized by anti-DimLe^x^ mAb SH2 by titrations and competitive inhibition experiments using oligosaccharide fragments of DimLe^x^ as well as Le^x^ analogues. We compare our results with that reported for anti-Le^x^ mAb SH1 and anti-polymeric Le^x^ mAbs 1G5F6 and 291-2G3-A. While SH1 recognizes an epitope localized to the non-reducing end Le^x^ trisaccharide, SH2, 1G5F6, and 291-2G3-A have greater affinity for DimLe^x^ conjugates than for Le^x^ conjugates. We show, however, that the Le^x^ trisaccharide is still an important recognition element for SH2, which (like 1G5F6 and 291-2G3-A) makes contacts with all three sugar units of Le^x^. In contrast to mAb SH1, anti-polymeric Le^x^ mAbs make contact with the Glc*N*Ac acetamido group, suggesting that epitopes extend further from the non-reducing end Le^x^. Results with SH2 show that this epitope is only recognized when DimLe^x^ is presented by glycoconjugates. We have reported that DimLe^x^ adopts two conformations around the β-d-Glc*N*Ac-(1→3)-d-Gal bond connecting the Le^x^ trisaccharides. We propose that only one of these conformations is recognized by SH2 and that this conformation is favored when the hexasaccharide is presented as part of a glycoconjugate such as DimLe^x^-bovine serum albumin (DimLe^x^-BSA). Proper presentation of the oligosaccharide candidate via conjugation to a protein or lipid is essential for the design of an anti-cancer vaccine or immunotherapeutic based on DimLe^x^.

## 1. Introduction

One molecular hallmark of cancer is aberrant glycosylation that results from abnormally expressed glycosyltransferases and glycosidases in tumor cells [1]. Cancer cells display glycans at different levels and profiles than normal cells [2,3]. The overexpression of these tumor associated carbohydrate antigens (TACAs) is frequently correlated with poor prognosis, allowing TACAs to be diagnostic markers [4]. In addition, there is mounting evidence that the overexpression of TACAs correlates with various stages of cancer, and that they play an important role in cancer proliferation, tumor cell metastasis, and invasiveness [5,6]. Thus, TACAs are of considerable interest in the search for anti-cancer immunotherapeutics, particularly since they may allow the differentiation between tumor and normal cells [1].

Amongst the many TACAs that have been characterized, several papers have reported the accumulation of fucose-containing glycosphingolipids in adenocarcinomas [7,8,9]. Of particular interest is the glycolipid displaying the dimeric Lewis X (DimLe^x^) hexasaccharide, which is reported to accumulate in colonic and liver adenocarcinomas and is associated with the progression of colorectal cancer [3,8,10]. In normal tissues, most of the type 2 chains (i.e., Gal-β-(1→4)-Glc*N*Ac linkage) are branched by β-(1→6)-Glc*N*Ac transferase [10,11,12]. However, tumor tissues undergo blocked synthesis of the branched lactosamine, synthesizing unbranched type 2 chains. These unbranched structures, upon straight chain elongation, undergo increased fucosylation and/or sialylation. The accumulation of DimLe^x^ on tumor tissues is a result of the enhanced activity of the β-(1→3)-Glc*N*Ac transferase and increased fucosylation [10,11,12]. Numerous monoclonal antibodies (mAbs) directed against various cancers (gastric cancer, colonic and small cell adenocarcinomas, lung squamous carcinoma) and cancer cell lines (leukemia HL-60, SCLC) were shown to react with the Lewis X (Le^x^) determinant similarly to a mAb directed against the stage-specific embryonic antigen-1 (SSEA-1) [11,13,14,15,16,17,18,19,20,21,22,23,24,25,26]. Antibodies directed against the Le^x^ determinant (anti-SSEA-1, FH3, etc.) were also shown to react within a number of normal tissues and cells (granulocytes, erythrocytes, colon mucosa, liver, etc.) [11,17,19,23,24,26]. In contrast, mAbs directed against di- and trimeric Le^x^ structures, such as FH4 and ACFH18, were found to react more specifically with certain tumor cell lines [25] and cancer tissues [27]. These results suggest that mAbs, such as ACH18 and FH4, are selective for internal epitopes presented by polymeric Le^x^ structures, while anti-monomeric Le^x^ mAbs (anti-SSEA-1, FH3 etc.) react with the terminal Le^x^ trisaccharide presented by mono-, di- and trimeric Le^x^ glycolipids on cancer cells and tissues [11,25]. Of particular interest to us are mAbs SH1 and SH2, which were raised upon immunization of mice with the Le^x^ glycolipid (III^3^FucnLc_4_) and DimLe^x^ glycolipid (III^3^V^3^Fuc_2_nLc_6_), respectively [3]. Much like mAbs anti-SSEA-1 and FH3, SH1 was shown to react with all fucosylated type 2 glycolipids displaying the Le^x^ trisaccharide at their non-reducing end. In contrast, much like mAb FH4, mAb SH2 shows a strong preference for di- and tri- fucosylated type 2 chains (III^3^V^3^Fuc_2_nLc_6_ and III^3^V^3^VII^3^Fuc_3_nLc_8_) displaying DimLe^x^ and TrimLe^x^, while it does not react with the monofucosylated Le^x^ glycolipid (III^3^FucnLc_4_) [3]. SH1 and SH2 were used to demonstrate increased levels of Le^x^ and DimLe^x^ glycolipids and glycoproteins in the sera of patients suffering from various types of adenocarcinomas [3].

Anti-monomeric Le^x^ and anti-multimeric Le^x^ mAbs have also been isolated upon infection of mice with *Schistosoma mansoni*. Indeed, it has been shown that mono- and polymeric Le^x^ glycolipids are expressed at various life stages of the parasite. Studies have illustrated that different oligomeric presentations of the Le^x^ antigen gave rise to the production of different groups of anti-Le^x^ mAbs in mice [28,29,30,31]. Depending on their binding profile to human serum albumin (HSA) glycoconjugates, Van Roon et al. proposed a classification of anti-Le^x^ mAbs into three groups: group I binds mono-, di-, and trimeric Le^x^ conjugates, group II binds di- and trimeric Le^x^ conjugates, and group III specifically binds the trimeric Le^x^-HSA conjugate [28].

Another anti-Le^x^ mAb (1G5F6) was cloned upon immunization of mice with the Gram-negative bacterial pathogen *Helicobacter pylori O:3* cells [32]. The cell envelope of *H. pylori* O-3 lipopolysaccharide (LPS) O-specific antigen (*O*-chain) were shown to be abundant in both Le^x^ and Le^y^ blood group epitopes [33,34]. The mAb 1G5F6 (IgG3) was found to recognize polymeric Le^x^ structures with greater affinity than monomeric Le^x^, suggesting that it recognizes epitopes that are either extended from the terminal non-reducing end Le^x^ or internal to DimLe^x^ [32]. Indeed, titration experiments that we recently reported [35] support that mAb 1G5F6 has greater affinity for a DimLe^x^ glycoconjugate than for a Le^x^ glycoconjugate. However, since mAb 1G5F6 retains binding to monomeric Le^x^, we propose to add a new group to Van Roon’s classification: [28] group IIB includes those mAbs that have greater affinity towards polymeric Le^x^ structures but still retain some binding to monomeric Le^x^.

In the past, we have extensively studied the group I anti-Le^x^ mAb SH1 [36,37]. In this paper we focus our studies on the murine IgG3 mAb SH2, which, as mentioned above, was raised in mice immunized with the purified DimLe^x^ glycolipid coated on *Salmonella Minnesota* [3]. SH2 was shown to react strongly with di- and trimeric Le^x^ glycolipids, while it does not bind to the monomeric Le^x^ ceramide pentasaccharide (LNFPIII) [3]. Thus, these preliminary studies suggest that SH2 is a group II anti-Le^x^ mAb as per the classification introduced earlier. For this reason, it is of interest to characterize the mAb, as this will provide insight into the internal epitopes displayed by DimLe^x^ on cancer cells.

## 2. Materials and Methods

### 2.1. Ascites Containing mAb SH2

Ascites containing mAb SH2 aliquots were a generous gift from S.-I. Hakomori from the Pacific Northwest Research Institute. In brief, immunization of BALB/c mice with Le^x^ pentasaccharide and DimLe^x^ glycolipids coated on *Salmonella Minnesota* was followed by the fusion of spleen cells with mouse Sp2 myeloma cells and the screening of antibody-secreting hybridomas by automated fluorescence immunoassay using mono- and dimeric Le^x^ glycolipids. Clone SH2 was selected and analyzed to be an IgG3 [3].

### 2.2. Preparation of the GDimLex-BSA (5) Glycoconjugate

The synthesis of the GDimLe^x^ cysteamine derivatives was previously reported [38]. The hexasaccharide was desalted on Dowex OH^−^. A solution (39 µL of 10 μL/mL, 1 equiv.) of 3,4-diethoxy-3-cyclobutene-1,2-dione (diethyl squarate) (Sigma Aldrich) in freshly distilled MeOH was added to a solution of the desalted hexasaccharide (2.9 mg, 2.5 μmol), in freshly distilled MeOH (300 μL). The reaction mixture was left at room temperature (RT) (4–6 h), and thin layer chromatography (TLC) (5:3:1 iPrOH-NH_4_OH-H_2_O) showed that the carbohydrate was quantitatively converted to the desired squarate adduct. Following concentration to dryness, the squarate adduct was solubilized in pH 10 carbonate buffer (100 µL, 0.1 M). The solution was transferred to a tube containing bovine serum albumin (BSA, 5.8 mg). The flask that contained the squarate solution was washed with more buffer, which was added to the reaction mixture (final volume of 300 μL). The reaction was left to proceed for 9 days at RT. The glycoconjugate was filtered against Milli-Q (MQ) H_2_O (7 × 8 mL) using an Amicon ultrafiltration cell equipped with a Diaflo membrane (Millipore, 25 mm, 30 kDa cut-off). The conjugate was then lyophilized to give the pure glycoconjugate: GDimLe^x^-BSA **5** (7.2 mg). The level of incorporation of the hexasaccharide to BSA was evaluated by MALDI-TOF (positive mode, matrix: sinapic acid) [39], which gave a hapten loading (n) of 16 GDimLe^x^ hexasaccharide per BSA (m/z: 86835).

### 2.3. Indirect Titration ELISA Procedures

MaxiSorp NUNC 96-well enzyme-linked immunosorbent assay (ELISA) microtiter plate (Thermo Fisher Scientific) was coated with a dilution of glycoconjugates **1**–**5** and BSA (100 μL per well, 10 μg/mL or 5 µg/mL as indicated in Figure 2) in a 10 mM phosphate-buffered saline (PBS) solution at pH 7.1. The plate was covered with sealing tape and incubated at 4 °C overnight. The antigen solution was discarded, and the plate was washed (using ELx405 auto plate washer, 5 × 15 s) with a 10 mM PBS buffer at pH 7.3 containing 0.05% Tween 20. The plate was blocked with 0.05% skim milk in 10 mM PBS (300 μL per well) and incubated for 1 h at 37 °C. The plate was then washed with 10 mM PBS-0.05% Tween 20. A 1:100 dilution of SH2 ascites was prepared and 146 μL of the dilution was distributed in the wells corresponding to the primary dilution. All other wells received 100 μL of the 10 mM PBS-0.05% Tween 20 pH 7.3 buffer. In-plate serial dilutions were performed in which 46 μL of the primary dilution was pipetted downward along the rows. The well contents were mixed by rinsing the pipette tips (7×). Lastly, 46 μL of the mAb solution was removed and discarded from the wells, which received the final solution of mAb (final volume in all wells, 100 μL). The last row of the 96-well plate was used as the blank control. MAb SH2 ascites were not pipetted into these wells, rather 100 μL of 10 mM PBS-0.05% Tween 20 was added. The plate was incubated for 2 h at 23 °C (in the dark). The plate was subsequently washed using the plate washer with PBS-0.05% Tween 20. A dilution of commercially available horseradish peroxidase labeled goat anti-mouse antibody (Mandel Scientific) (1:5000 in 10 mM PBS-0.05% Tween 20, 100 μL per well) was added to each well. After 1 h of incubation at 23 °C (in the dark), the plate was washed with PBS-0.05% Tween 20. A solution of the chromogenic substrate 3,3′,5,5′-tetramethylbenzidine (TMB) (Mandel Scientific) (100 μL per well) was added. After a 10 min incubation period at 23 °C (in the dark), 100 μL of 5% Phosphoric Acid stop solution was added to the wells to quench the reaction. The absorbance values were read at 450 nm employing a PowerWave XS plate reader. All samples were prepared in triplicate. The absorbance values were plotted against an increasing serum dilution. The values were fitted to a 4-parameter logistics sigmoidal equation: *y* = *y_0_* + *a*/[1 + (*x*/*x_0_*)*^b^*] using Sigma Plot^®®^ 10.0.

### 2.4. Competitive ELISA Procedures

A MaxiSorp NUNC 96-well ELISA microtiter plate was coated with a dilution of the (DimLe^x^)_16_-BSA conjugate **1** (100 μL per well, 5 μg/mL) in a 10 mM PBS solution at pH 7.1. The plate was covered with sealing tape and incubated at 4 °C overnight. The antigen solution was discarded, and the plate was washed (using ELx405 auto plate washer, 5 × 15 s) with a 10 mM PBS buffer at pH 7.3 containing 0.05% Tween 20. In 1.5 mL Eppendorf tubes, 175 μL dilutions of competitors (concentration ranging from 0.632 mg/mL to 2 mg/mL in 10 mM PBS-0.05% Tween 20) were incubated for 1 h at 23 °C (in the dark) with 175 μL of a solution of SH2 ascites (1:500 dilution in 10 mM PBS-0.05% Tween 20). At the same time, the plate was blocked with 0.05% skim milk in PBS (300 μL per well). After 1 h of incubation at 37 °C, the plate was washed with 10 mM PBS-0.05% Tween 20. Each well then received 100 μL of the competitor-mAb mixture. Three wells were used as the blank control in which the competitor-mAb mixture was not added, rather received 100 μL of 10 mM PBS-0.05% Tween 20. The plate was incubated for 3 h at 23 °C (in the dark). The plate was subsequently washed using the plate washer with PBS-0.05% Tween 20. A dilution of commercially available horseradish peroxidase labeled goat anti-mouse antibody (1:5000 in 10 mM PBS-0.05% Tween 20, 100 μL per well) was added to each well. After 1 h of incubation at 23 °C (in the dark), the plate was washed with 10 mM PBS-0.05% Tween 20. A solution of the chromogenic substrate TMB (100 μL per well) was added. After a 10 min incubation period at 23 °C (in the dark), 100 μL of 5% Phosphoric Acid stop solution was added to the wells to quench the reaction. The absorbance values were read at 450 nm employing a PowerWave XS plate reader. All samples were prepared in triplicate. The absorbance values were plotted as the percentage inhibition against an increasing concentration of competitor, calculated using wells containing no competitor as the reference point. The values were fitted to a 4-parameter logistics sigmoidal equation: *y* = *y_0_* + *a*/[1 + (*x*/*x_0_*)*^b^*] using Sigma Plot 10.0. The concentration of each analogue required for 50% inhibition (IC_50_) of Le^x^-SH2 binding and the corresponding changes in free energy Δ(ΔG) of binding (kcal·mol^−1^) using DimLe^x^OPr **6** (Table 1) or Le^x^OMe **7** (Table 2) as the reference were calculated.

## 3. Results

### 3.1. Titration Experiments

**Figure 1:**
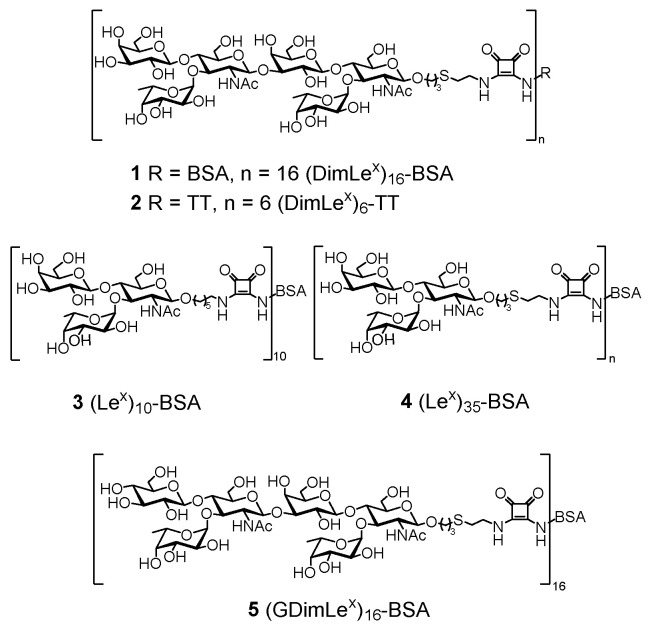
Glycoconjugates used in the titrations.

The ascites of SH2 were titrated against five glycoconjugates shown in Figure 1: (DimLe^x^)_16_-BSA **1**, (DimLe^x^)_6_-TT **2**, (Le^x^)_10_-BSA **3**, (Le^x^)_35_-BSA **4**, and (GDimLe^x^)_16_-BSA **5** as well as BSA.

**Figure 2:**
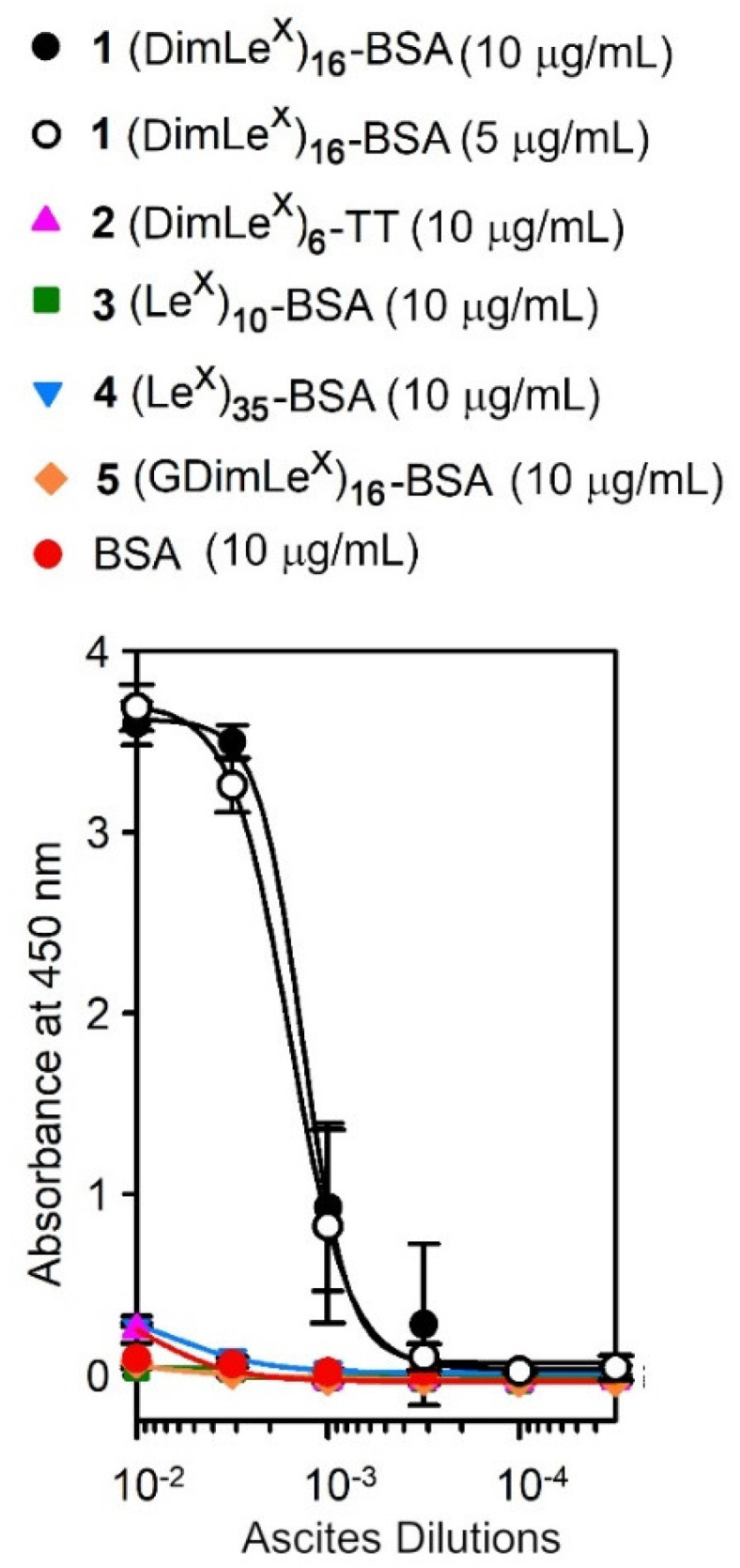
Titration curves for SH2 ascites with conjugates **1**–**5** and BSA.

The preparations of glycoconjugates **1**–**4** have been described previously, Refs. [35,36] and the preparation of glycoconjugate **5** is described above. All antigens were coated at a concentration of 10 μg/mL, with the exception of (DimLe^x^)_16_-BSA (**1**), which was coated at both 10 μg/mL and 5 µg/mL (Figure 2). Titration curves were generated by plotting the absorbance measured at 450 nm against varying dilutions of SH2, and the curves were fitted to a four-parameter logistics sigmoidal equation (Figure 2).

The titration against BSA confirmed that mAb SH2 had no affinity for the carrier protein and that the results obtained with the glycoconjugates resulted from the ability for SH2 to bind to the oligosaccharide. As expected from the initial study by Singhal et al. [3], the mAb SH2 showed high specificity for the (DimLe^x^)_16_-BSA glycoconjugate **1** whether the plate was coated with a 10 or 5 µg/mL solution of the conjugate (EC_50_ of ~ 1:600 for both concentrations). However, coating with a 3 µg/mL concentration of the DimLe^x^)_16_-BSA conjugate (see Figure S1) gave a titration curve that did not reach maximum optical density (OD). In contrast, the mAb displayed only very little binding to (DimLe^x^)_6_-TT **2** even when using higher coating concentration of 20 µg/mL (not shown). This lack of binding suggests that either the hexasaccharide is sterically hindered and not accessible to the mAb when on tetanous toxoid (TT), or that the presentation of the antigen by TT is inadequate for binding by SH2 [40,41,42]. In contrast to anti-Le^x^ mAbs SH1 and 1G5F6 [35,36], SH2 displayed no binding to conjugate (Le^x^)_10_-BSA **3** (Figure 2, green). We tested whether the lack of recognition to glycoconjugate **3** was the result of avidity, by immobilizing the (Le^x^)_35_-BSA glycoconjugate **4**. From Figure 2, it can be seen that SH2 only weakly binds to glycoconjugate **4** (blue), portraying a binding curve similar to (DimLe^x^)_6_-TT **2**.

Our previous study of the anti-Le^x^ mAb SH1 showed that replacing the galactose residue in Le^x^ by a glucose unit (GlcLe^x^) resulted in a total loss of binding by SH1 [36]. Thus, we had postulated that a DimLe^x^-based vaccine candidate that may elicit group II and III antibodies but not elicit the production of group I anti-Le^x^ antibodies could be an analogue of DimLe^x^ in which the non-reducing end galactose unit was replaced by glucose (GDimLe^x^). To test the hypothesis that such an analogue would be recognized by group II/III type antibodies, we titrated SH2 against immobilized the conjugate (GDimLe^x^)_16_-BSA **5**. Unfortunately, SH2 displayed no binding to (GDimLe^x^)_16_-BSA even with a higher coating concentration (20 μg/mL, not shown) of the glycoconjugate. The weak recognition of glycoconjugates Le^x^-BSA **3** and **4** suggests that SH2 binds an internal epitope of DimLe^x^ that involves the non-reducing end galactosyl residue. Hence, we attempted to map this internal epitope using competitive inhibition experiments with various soluble fragments of the hexasaccharide.

### 3.2. Competitive Inhibition Studies with DimLe^x^ Fragments and Glycoconjugates

**Figure 3:**
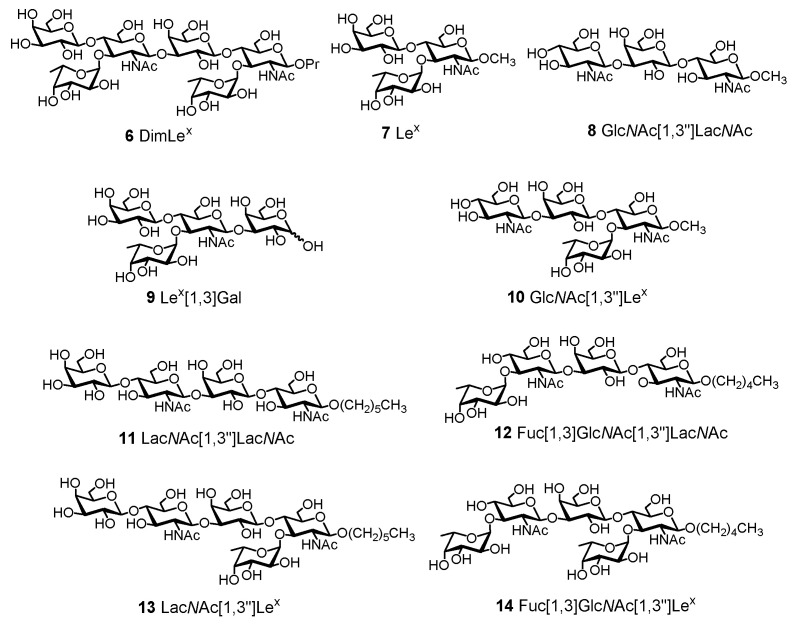
Structure of inhibitors **6**–**14** used in this study.

We have previously reported the chemical synthesis of the DimLe^x^ propyl hexasaccharide **6** as well as that of the Le^x^ methyl glycoside **7** [38,43]. In addition, we have also described the synthesis of tri-, tetra-, and pentasaccharide fragments of DimLe^x^ **8**, **10**–**14** [44,45,46,47] shown in Figure 3. Tetrasaccharide fragment 9 (Lex[1,3]Gal, Figure 3) was a generous gift of Samain and co. [48].

**Figure 4:**
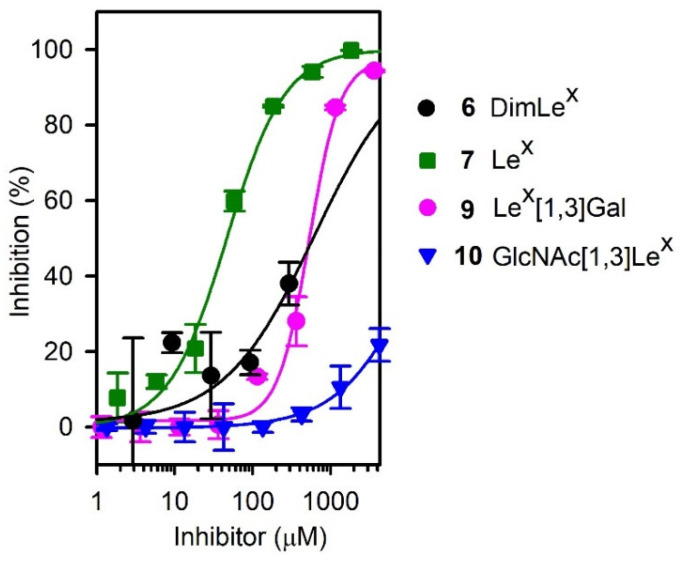
Competitive inhibitions curves for those compounds **6**, **7**, **9**, **10** that showed some inhibition. Coated inhibitor (DimLe^x^)_16_-BSA (**1**) and SH2 serum dilution 1:500.

The affinity of mAb SH2 for DimLe^x^ **6** was compared to its affinity for this panel of DimLe^x^ fragments **6**–**14**. We performed competitive ELISA experiments using DimLe^x^-BSA **1** (coated at 5 μg/mL) as an immobilized antigen, DimLe^x^ fragments **6**–**14** as soluble competitors, and SH2 ascites dilutions of 1:500. Figure 4 shows the inhibition curves only for those compounds **6**, **7**, **9**, **10** that showed some inhibition, and Table 1 lists the corresponding IC_50_ values. Table 1 also shows the changes in free energy of binding [Δ(ΔG)] for fragments **7**, **9**, **10** that were calculated using DimLe^x^ **6** as a reference.

In addition to DimLe^x^ (**6**), only three fragments: Lex (7), LexGal (9) and GlcNAc[1,3″]Lex (10), albeit weakly, were shown to inhibit the DimLex-SH2 binding. The best inhibitors: DimLex (6) Lex (7) and Lex[1,3]Gal (10) all contain the terminal non-reducing end Lex trisaccharide, which is absent in all other fragments. Indeed, both fucosyl and galactosyl residues in the non-reducing end Lex trisaccharide are required for inhibition, as is shown by the lack of competitive binding of the LacNAc[1,3″]Lex (13) and Fuc[1,3,]GlcNAc[1,3″]Le^x^ (**14**) pentasaccharide fragments. Most surprisingly, our inhibition results showed that all compounds, including the native DimLe^x^ antigen **6** were weaker inhibitors than the Le^x^ trisaccharide **7** (Table 1). In fact, the DimLe^x^ hexasaccharide 6 and the Lex[1,3]Gal tetrasaccharide **9** gave similar values of IC_50_ (~500 µM, Table 1, entries 1 and 3), while the Le^x^ trisaccharide **7** had greater binding affinity with an IC_50_ of 47 µM, resulting in a greater binding to SH2 by a Δ(ΔG) of -1.4 kcal.mol^−1^ when compared to DimLe^x^ **6** (Table 1, entry 2). These results are surprising since both hexasaccharide **6** and tetrasaccharide **9** display the reducing end Le^x^ trisaccharide, which we would expect binding to SH2 with the same extent than the Le^x^ trisaccharide **7**. These results also come in sharp contrast with the very little to no binding of SH2 to the Le^x^-BSA conjugates **3** and **4** in our initial titration experiments (Figure 2). Thus, it appears that presentation of the Le^x^ trisaccharide and DimLe^x^ hexasaccharide has an impact on their ability to be recognized by SH2.

**Figure 5:**
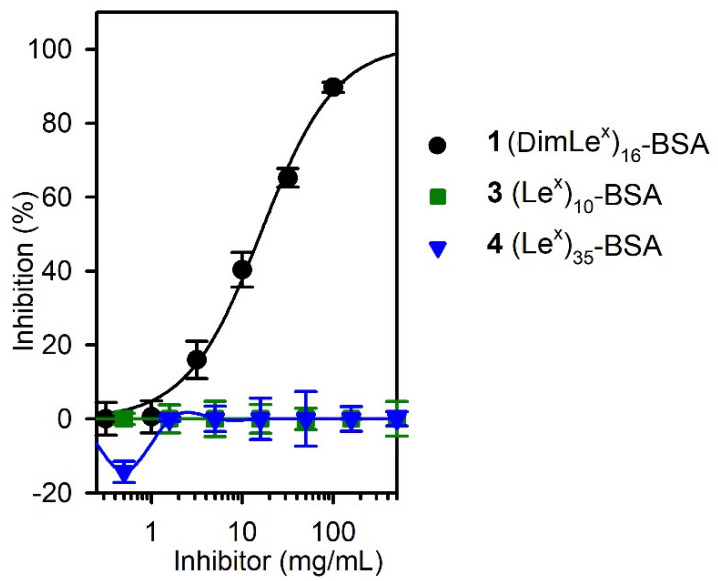
Competitive inhibition with glycoconjugates **1**, **3**, and **4** as soluble inhibitors. Coated inhibitor (DimLe^x^)_16_-BSA (**1**). SH2 serum dilution, 1:500.

To further confirm the results of our titration experiments, we carried out competitive inhibition experiments using the (DimLe^x^)_16_-BSA **1** as the immobilized antigen and the conjugates (DimLe^x^)_16_-BSA (**1**), (Le^x^)_10_-BSA (**3**), and (Le^x^)_35_-BSA (**4**) as soluble inhibitors. Indeed, as expected, only the DimLe^x^ conjugate **1** was able to inhibit binding, while the Le^x^ conjugates did not (Figure 5).

### 3.3. Competitive Inhibition Binding Studies with Le^x^ Analogues

**Figure 6:**
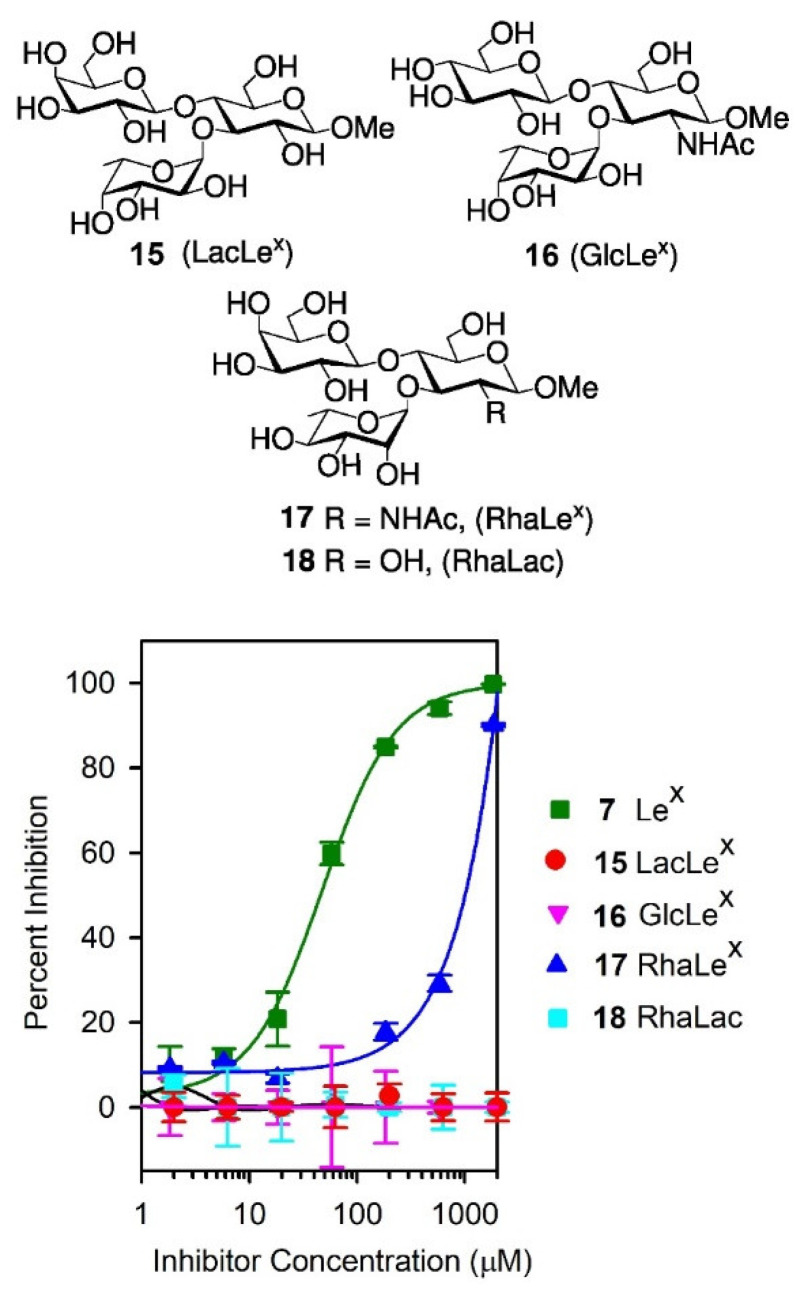
Structures of analogue **15**–**18** and competitive inhibition with **7** and **15**–**18** as soluble inhibitors. Coated inhibitor (DimLe^x^)_16_-BSA (**1**). SH2 serum dilution, 1:500.

Given the surprising ability of the Le^x^ trisaccharide **7** to inhibit the binding of SH2 to (DimLe^x^)_16_-BSA **1**, we also investigated the inhibition of this binding by our previously reported [43,49] Le^x^ analogues **15**–**18** (Figure 6). While D-Glc*N*Ac and D-Gal are replaced by a D-glucose unit in analogue **15** (LacLe^x^) and **16** (GlcLe^x^), respectively, the L-fucose residue is replaced by an L-rhamnose unit in analogue **17** (RhaLe^x^). In analogue **18**, both the D-Glc*N*Ac and L-Fuc are replaced by D-glucose and L-rhamnose units, respectively. Our previous studies [36] have shown that such analogues maintain the typical stacked conformation of the Le^x^ trisaccharide [36,50,51,52,53,54]. We performed competitive inhibitions experiments using the (DimLe^x^)_16_-BSA conjugate **1** (5 μg/mL) as the immobilized ligand and analogues **15**–**18** as soluble competitors. Figure 6 shows the corresponding inhibition curves, while Table 2, entries 2–5, gives the IC_50_ for each analogue and the corresponding changes in free energy of binding Δ(ΔG) taking Le^x^ **7** as the reference inhibitor (entry 1).

As can be seen, LacLe^x^ **15**, GlcLe^x^ **16**, and RhaLac **18** showed no inhibition of binding. The lack of inhibition of analogues **15** (LacLe^x^) and **18** (RhaLac) indicates the importance of the *N*-acetylglucosamine residue, suggesting that the amide group participates in an essential antibody–carbohydrate interaction. Moreover, since replacing the galactose unit by a glucose residue also resulted in a complete loss in binding to GlcLe^x^ **16**, we identified the galactose unit as a crucial element in epitope recognition by the mAb. This later result was in agreement with the titrations experiments performed with (GDimLe^x^)_16_-BSA that showed no binding of SH2 (Figure 2). In contrast, substitution of the fucose unit by a rhamnose residue in RhaLe^x^ (**17**) resulted only in a 1.7 kcal.mol^−1^ decrease in free energy of binding compared to the Le^x^ antigen **7** (Table 2, entry 4). This loss of binding correlates with the loss of a key polar interaction or H-bond, occurring between either Fuc 2-OH or 4-OH and an amino acid side chain in the SH2 binding site [55,56].

**Figure 7:**
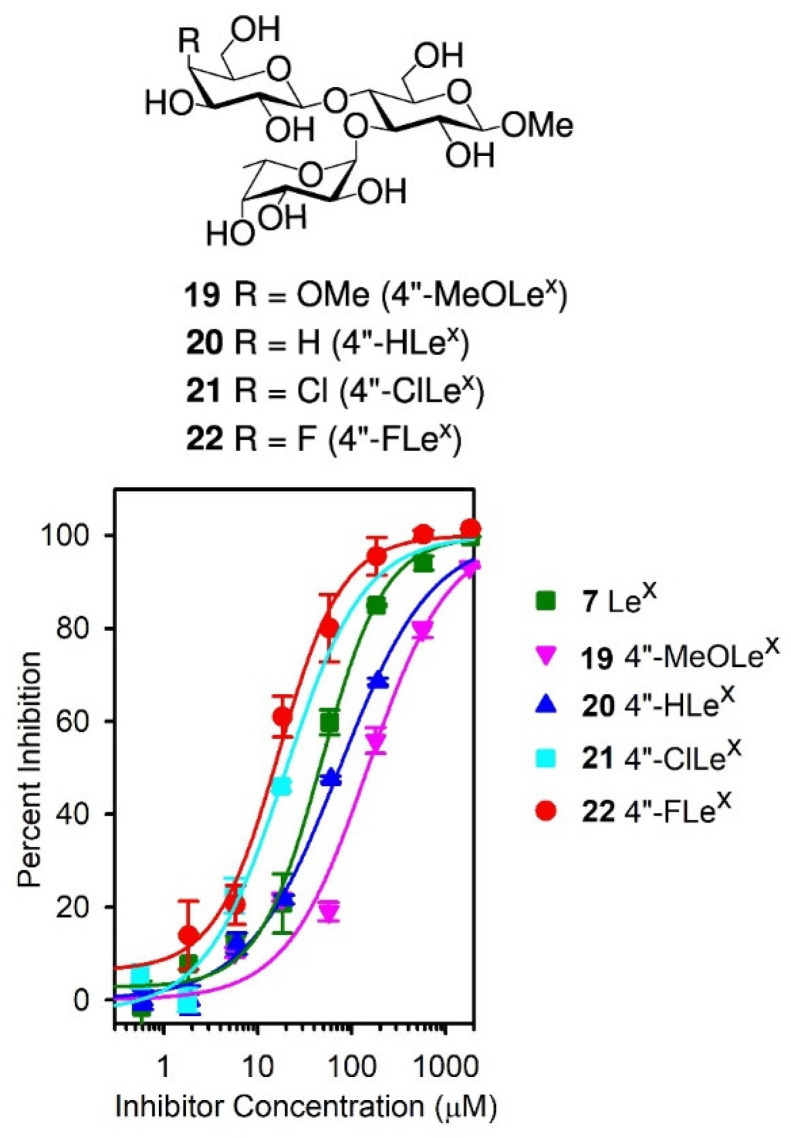
Structures of analogue **19**–**22** and competitive inhibition with **7** and **19**–**22** as soluble inhibitors. Coated inhibitor (DimLe^x^)_16_-BSA (**1**). SH2 serum dilution, 1:500.

Given the lack of inhibition observed for GlcLe^x^ **16** and the lack of binding of the (GDimLe^x^)_16_-BSA conjugate in our titration experiments (Figure 2), we investigated the importance of the galactosyl 4-OH group for recognition by SH2 using the previously described [57] analogues **19**–**22** in competitive binding experiments. In these analogues, the galactosyl 4-OH is either methylated (4″-MeOLe^x^, **19**), deoxygenated (4″-HLe^x^, **20**), or replaced by a halogen in the 4″-ClLe^x^ (**21**) and 4″-FLe^x^ (**22**) (Figure 7).

Accordingly, we carried out competitive inhibitions experiments using the (DimLe^x^)_16_-BSA conjugate **1** (5 μg/mL) as the immobilized ligand and analogues **19**–**22** as soluble competitors. Figure 7 shows the corresponding inhibition curves, while Table 2, entries 6–9, gives the IC_50_ values for each analogue and the corresponding changes in free energy of binding Δ(ΔG) taking Le^x^ **7** as the reference inhibitor. As can be seen in Figure 7, all analogues modified at O-4″ were able to inhibit the (DimLe^x^)_16_-BSA binding to SH2 almost as well as or better than the Le^x^ trisaccharide **7**. Compared to Le^x^ **7**, the 4″-MeOLe^x^ analogue **19** resulted only in a small 0.7 kcal.mol^−1^ decrease in free energy of binding, suggesting that the galactosyl 4-OH is partially solvent exposed (Table 2, entry 6) [56]. Since the 4″-deoxy analogue **20** (4″HLe^x^) only resulted in a loss of 0.3 kcal.mol^−1^ binding energy (Table 2, entry 7), it appears that the galactose 4-OH is not involved in any strong polar interaction or H-bond within the SH2 binding site [56]. Finally, the slight increases in binding of −0.5 and −0.6 kcal.mol^−1^ for the 4″-halogenated analogues **21** (4″-ClLe^x^) and **22** (4″-FLe^x^) suggest that the 4″-OH galactose is involved in weak van der Waals interactions. Indeed, this enhanced binding reflects the ability of the halogens (Cl and F) to produce stronger van der Waals forces than the hydroxyl group [58].

## 4. Discussion

**Figure 8:**
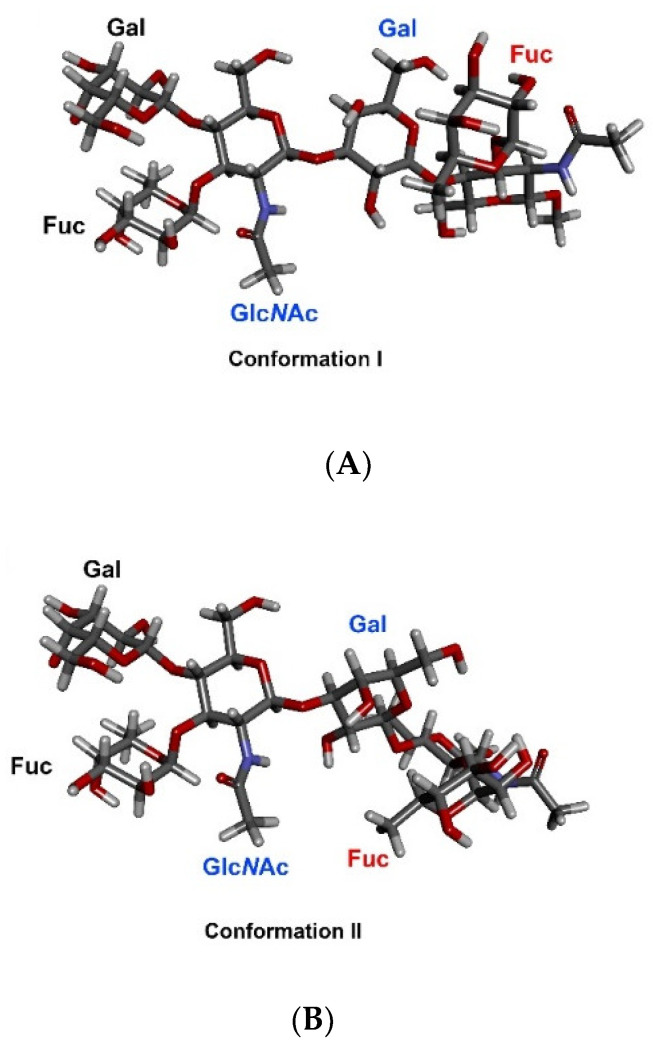
Known [66] conformations of the DimLe^x^ hexasaccharide in fast exchange: (**A**) Conformation I; (**B**) Conformation II.

MAb SH2 is shown here to bind (DimLe^x^)_16_-BSA (**1**) but not (DimLe^x^)_6_-TT (**2**) nor to the (Le^x^)_10_-BSA conjugate (**3**). Thus, these results seem to indicate that SH2 is binding an internal epitope on DimLe^x^, which is not displayed by the Le^x^-BSA conjugate and not accessible on the DimLe^x^-TT conjugate possibly as a result of the bulky protein carrier or inadequate presentation by the protein [40,41,42]. However, we also report here that the binding of SH2 to DimLe^x^-BSA is inhibited better by Le^x^ trisaccharide (**7**) than by DimLe^x^ hexasaccharide (**6**). This latter result contrasts with the hypothesis that SH2 binds an internal epitope on DimLe^x^. One might propose that the SH2 mAb binding site is a deep pocket, which does not allow proper binding to the Le^x^ trisaccharide when displayed on BSA due to steric hindrance, while it would be more accessible on the (DimLex)16-BSA conjugate. If this was so, one would then expect equivalent inhibition by all soluble inhibitors that display the reducing end Lex, such as DimLex (6), Lex (7) and Lex[1,3]Gal (9). This was not the case, since DimLex (6) and Lex[1,3]Gal (**9**) were much weaker inhibitors than the Le^x^ trisaccharide (**7**). Thus, we propose that recognition by mAb SH2 of the epitope displayed by the DimLe^x^ antigen involves the reducing end Le^x^ trisaccharide extending further to part of the reducing end Le^x^ trisaccharide. However, binding of this epitope by SH2 is subject to correct presentation by the carrier molecule. Indeed, it has been shown that the recognition of oligosaccharides by lectins, enzymes, and antibodies was influenced by the different geometries of presentation of the carbohydrate within the glycan-carrier system (i.e., glycoconjugates, glycoproteins, glycolipids) [40,41,42,59]. Extended epitope presentation from the non-reducing end Le^x^ trisaccharide in DimLe^x^ to the mAb SH2 binding site should also be considered in the context of the β-d-Glc*N*Ac-(1→3)-d-Gal glycosidic bond conformation. Indeed, this glycosidic bond, that links the non-reducing end Le^x^ trisaccharide to the reducing end Le^x^ moiety in DimLe^x^ (**6**) or to galactose in Lex[1,3]Gal (9), has been shown to be highly flexible in various oligosaccharides [60,61,62,63,64,65,66]. Conformations around glycosidic bonds are defined by two dihedral angles: Φ (O5-C1-O1-Cx) and Ψ (C1-O1-Cx + 1). While it has been well established that the Le^x^ trisaccharide adopts a rigid “stacked” conformation [36,50,51,52,53,54], we have shown [66] that the DimLe^x^ hexasaccharide could adopt two distinct conformations (I and II) around the Ψ dihedral angle for the β-d-Glc*N*Ac-(1→3)-d-Gal glycosidic bond (Figure 8). These conformations were shown by NMR to exist in fast exchange for the hexasaccharide in solution [66].

In conformation I (Figure 8A), the non-reducing end β-Glc*N*Ac (Glc*N*Ac labelled in blue) unit is in the same plane as the reducing end galactosyl unit (Gal labelled in blue), while in conformation II, these two sugar units are perpendicular to one another (Figure 8B). Thus, these two conformations result in a much different environment around the *N*-acetyl group of the non-reducing end β-Glc*N*Ac, which ends up in much closer proximity to the reducing end fucose unit (Fuc labelled in red) in conformation II than in conformation I. Taking together the importance of epitope presentation for binding of SH2 to Le^x^ and DimLe^x^ discussed above and the occurrence of two distinct conformations of the DimLe^x^ hexasaccharide (and analogues) in solution, we propose that one of these conformations impedes the binding of SH2 to the non-reducing end Le^x^ trisaccharide. This explains the greater affinity of SH2 for the Le^x^ trisaccharide **7** than for the DimLe^x^ hexasaccharide **6** and the Lex[1,3]Gal fragment **9**. We propose that immobilization on BSA in the (DimLe^x^)_16_-BSA conjugate **1** favors one conformation of the DimLe^x^ oligosaccharide that presents the correct epitope accessible for binding with SH2.

Our results with the analogues and fragments suggest that, pending proper presentation, the non-reducing end Le^x^ trisaccharide is an essential binding element for recognition by mAb SH2, despite the fact that SH2 does not react with the Le^x^-BSA conjugate nor with the Le^x^ ceramide pentasaccharide (LNFPIII) [3]. These results are interesting when compared to the results that we have already described for mAbs SH1 and 1G5F6. As mentioned before, murine mAb SH1 (IgG3) was raised against the monomeric Le^x^ ceramide pentasaccharide (LNFPIII) coated on acid-treated *Salmonella Minnesota*. It was shown to exhibit high affinity for monomeric and polymeric Le^x^ structures regardless of chain length and is therefore classified as an anti-Le^x^ group I mAb that binds the terminal non reducing end Le^x^ trisaccharide [3,28]. In contrast, the murine mAb 1G5F6 (IgG3) raised against *Helicobacter pylori* O:3, much like mAb SH2, was shown to recognized polymeric Le^x^ structures with greater affinity than monomeric Le^x^ [32,35]. However, since 1G5F6 still retains binding to the Le^x^ trisaccharide, we propose to classify it as a group IIB anti-Le^x^ mAb. We have previously studied the recognition of monomeric Le^x^ by anti-Le^x^ mAb SH1 and anti-polymeric Le^x^ mAb 1G5F6 [35,36,37]. For comparison with SH2, the difference in changes of free energy reported for competitive inhibition experiments with SH1 and 1G5F6 are reproduced in Table 2 taking the Le^x^ (**7**) trisaccharide as a reference. As can be seen, substitution of the β-D-Glc*N*Ac unit by the β-D-Glc greatly affected binding to SH2 and 1G5F6, (Table 2, entry 2) but not to SH1 [35,36]. Thus, in contrast to group I mAb SH1, the *N*-acetyl group of the Glc*N*Ac residue is an essential binding element for recognition by anti-polymeric Le^x^ mAbs SH2 and 1G5F6. Indeed, we have proposed—much like we do here for SH2—that 1G5G6 recognizes an epitope that, including the Le^x^ non-reducing end trisaccharide, extends towards the galactosyl ring of the reducing end Le^x^ trisaccharide. Replacing the galactose unit by a glucose residue led to total loss of recognition by both SH1 and SH2 and a loss of binding by 2.7 kcal.mol^−1^ for 1G5F6 [35,36]. Such reduced binding cannot solely be explained by the loss of favorable interactions between the binding sites of mAb SH1, SH2 or 1G5F6, and the axial galactose 4-OH group. Therefore, it is likely that the equatorial orientation of the 4″-OH group in GlcLe^x^ **16** disturbs the hydrophobic patch normally present in the β-galactosyl α face and leads to these results. Indeed, the β-galactosyl α face, which is defined by H-1, H-3, H-4, and H-5 of the galactose ring [67,68], is known to constitute an important recognition element due to its interaction with aromatic amino acid residues present in anti-carbohydrate antibodies and lectin binding sites [29,37,55,69]. Thus, as for mAbs SH1 and 1G5F6, we propose that the hydrophobic α-face of the galactose residue is probably involved in stacking interactions with aromatic side chains within the binding site [35,36,37]. Results with the RhaLe^x^ analogue **17** (Table 2, entry 4) showed that all three mAbs are involved in polar interactions or hydrogen bonds (H-bonds) involving the Fuc 2-OH and/or 4-OH with amino acid side chains in the binding sites. Noticeably, mAbs SH2 and 1G5F6 led to greater changes in free energy of binding [Δ(ΔG) = 1.6–1.7 kcal.mol^−1^] than mAb SH1 [Δ(ΔG) = 1.1 kcal.mol^−1^] suggesting that for these mAbs, both 2-OH and 4-OH of the fucosyl residue are involved in binding when only one of these is involved in binding to mAb SH1. Furthermore, for all three mAbs a cumulative effect was observed when both *N*-acetylglucosamine and fucose units were substituted by glucose and rhamnose residues (**18**, Table 2, entry 5), respectively (Table 2, entry 5). Taken together, these results illustrate that, while all three sugar residues are involved in the recognition of Le^x^ by mAbs SH2 and 1G5F6, mAb SH1 makes contacts with the galactosyl and fucosyl residues, while no interaction with the Glc*N*Ac acetamido group is detected.

Studies in the past have used methylated analogues of carbohydrates to distinguish between hydroxyl groups located at the periphery of mAb binding sites from those that are solvent exposed. It has been shown that partially solvent exposed hydroxyl groups can be replaced by methoxy groups with only a minor change in binding energy [70,71]. Thus, results with the 4″-MeOLe^x^ analogue **19** (Table 2, entry 6) indicate that, while the galactosyl 4-OH is likely positioned at the periphery or within the biding sites of mAbs SH1 and 1G5F6, it is partially exposed to bulk solvent in the mAb SH2 binding site. Results with the 4″-deoxy Le^x^ **20** (4″-HLe^x^, entry 7) show that, while the galactosyl 4-OH is likely involved in hydrogen bonding within the SH1 binding site [Δ(ΔG) = 1.3 kcal.mol^−1^], it does not contribute to the to the binding of Le^x^ to mAbs SH2 and 1G5F6. Similarly, while replacing the galactosyl 4-OH by a chlorine or fluorine (entries 8 and 9) results in large drops in free energy of binding (1.6 and 2.1 kcal.mol^−1^) with mAb SH1, these substitutions have relatively little impact on the binding to mAbs SH2 and 1G5F6. Thus, while this hydroxyl group acts as an H-bond donor within the SH1 binding site [37], it is located at the periphery of the binding site within the 1G5F6 binding site [35] and partially exposed to bulk solvent in the SH2 binding site only contributing weak polar contacts in the latter two cases. Collectively, these results emphasize the different roles of galactose in the recognition of Le^x^ by the three mAbs. In mAb SH1, the galactose participates in both hydrogen bond formation and hydrophobic interactions with the galactosyl α-face. In contrast, in mAbs SH2 and 1G5F6, the galactose predominantly participates through hydrophobic interactions with the galactosyl α-face.

Our results should be compared to the work of Van Roon et al. who studied anti-Le^x^ antibodies generated in mice infected with *Schistosoma mansoni* cercariae [28,29,72]. In their study, Van Roon et al. cloned mAb 291-2G3A (IgG3), which, similarly to SH2 and 1G5F6, was shown by surface plasmon resonance (SPR) studies to recognize a DimLe^x^-HSA conjugate with greater affinity than Lacto-N-fucopentaose-HSA (LNFPIII-HSA) conjugate [29,72]. To understand the specificity of the antibody for Le^x^, Van Roon et al. performed X-ray crystallographic analysis of the Fab fragment of mAb 291-2G3-A in complex with the Le^x^ trisaccharide. The binding site of mAb 291-2G3-A is described as a shallow pocket that, much like SH2 and 1G5F6, makes contact with all three sugar units of the Le^x^ trisaccharide. Again, similarly to SH2 and 1G5F6, the 4-OH of galactose does not participate in an H-bond with the mAb 291-2G3-A binding site, but a tryptophan (W33) residue forms favorable aromatic stacking interactions with the hydrophobic patch of the galactosyl α face [29]. Finally, the authors also report an H-bond between the glucosamine acetamido nitrogen and an asparagine residue (Asn L91) in the binding site. Unfortunately, the authors were not able to obtain a crystal structure with DimLe^x^ and thus could not conclude if binding to 291-2G3-A to DimLe^x^ would extend further from the reducing end Glc*N*Ac residue.

## 5. Conclusions

These results taken collectively clearly demonstrate that different groups of antibodies are produced depending on the presentation of Le^x^ to the immune system. Since SH1 was raised against the Le^x^ ceramide pentasaccharide (LNFPIII), it recognizes an epitope localized to the non-reducing end Le^x^ trisaccharide in all Le^x^-displaying analogues and conjugates. In contrast, those mAbs raised against polymeric Le^x^ structures, such as SH2, 1G5F6, and 291-2G3-A, have greater affinity for DimLe^x^ conjugates than for conjugates only displaying Le^x^. However, the results presented in this work for SH2, as well as those reported for 1G5F6 and 291-2G3-A, indicate that the non-reducing end Le^x^ trisaccharide is still an important recognition element for these mAbs [29,36,37]. Indeed, all three mAbs make similar contacts with all three sugar units of the Le^x^ trisaccharide including the galactosyl hydrophobic α face. Since all three mAbs (SH2, 1G5F6, 291-2G3-A) make contact with the Glc*N*Ac acetamido group and, given their higher affinity towards DimLe^x^ conjugates than for Le^x^ conjugates, we also conclude that they recognise an epitope that extends further from the non-reducing end Le^x^ trisaccharide. However, results with SH2 indicate that this extended epitope is only recognized when DimLe^x^ is presented by conjugates such as BSA glycoconjugates or glycolipids [3]. Indeed, while SH2 recognizes the DimLe^x^-BSA conjugate **1** but does not bind (or binds poorly) to the Le^x^-BSA conjugates **3** or **4** (Figure 1), it displays weaker binding to the DimLe^x^ propyl glycoside **6** than for the Le^x^ methyl glycoside **7**. Interestingly, the known flexibility of the β-d-Glc*N*Ac-(1→3)-d-Gal glycosidic bond that connects the two Le^x^ trisaccharides in DimLe^x^ results in the hexasaccharide adopting two conformations in fast exchange when in solution (Figure 8). Thus, it is reasonable to assume that only one of these conformations is recognized by SH2, and we propose that this conformation is favored when the hexasaccharide is presented as part of a glycolipid such as the DimLe^x^ glycolipid (III^3^V^3^Fuc_2_nLc_6_) or as glycoprotein such as DimLe^x^-BSA **1**. Given the steric hindrance observed around the Glc*N*Ac acetamido group in conformation II (Figure 8B), it is reasonable to suggest that conformation I (Figure 8B) is the conformation recognized by SH2.

**Figure 9:**
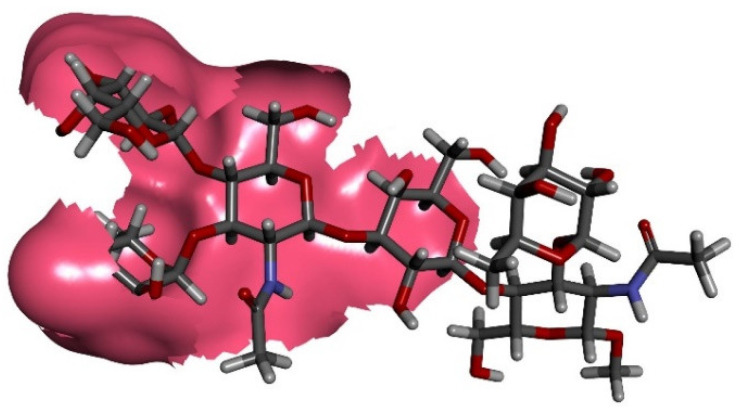
Proposed extended epitope recognized by SH2 in conformation I of the DimLe^x^ hexasaccharide.

Additional studies with mAb 1G5F6 and the analogues and fragments used in the present work will establish if presentation of DimLe^x^ also has an impact on epitope recognition by mAb 1G5F6. Based on the results reported here, we propose in Figure 9 a schematic representation of the epitope recognized by SH2 in conformation I of DimLe^x^.

While the DimLe^x^ antigen is almost exclusively expressed at the surface of tumor cells, this hexasaccharide displays at its reducing end the Le^x^ trisaccharide that is expressed on a number of normal tissues and cells. Thus, the development of anti-cancer vaccines able to target the DimLe^x^ antigen safely, while avoiding an autoimmune response against the Le^x^ antigen, requires that we identify and target epitopes that are presented by the DimLe^x^ hexasaccharide but not displayed by the Le^x^ trisaccharide. Such epitopes are recognized by mAbs such as SH2 and 1G5F6. Indeed, these two mAbs bind strongly to DimLe^x^ conjugates, while they do not, or very poorly, bind glycoconjugates displaying only the Le^x^ trisaccharide. Therefore, the development of anti-cancer vaccines able to target the DimLe^x^ antigen rests on the accurate mapping of epitopes recognized by mAbs such as SH2 and 1G5F6. Most important to the field of TACA-based anticancer therapies, the results reported here indicate that proper presentation of the DimLe^x^-based antigen target via conjugation to a protein or lipid is vital to the design of a successful vaccine or immunotherapeutic. Further mapping of this epitope will be explored using oligosaccharide analogues and fragments conjugated to BSA to ensure appropriate presentation of the epitope.

## Figures and Tables

**Table 1:** Inhibition data for mAb SH2 and those compounds **6**, **7**, **9**, **10** that showed some inhibition.

Entry	Inhibitor	IC_50_ *^a^* (μM)	∆(∆G) *^b^* (kcal·mol^−1^)
1	DimLe^x^ **(6)**	503	0
2	Le^x^ **(7)**	47	−1.4
3	Lex[1,3]Gal **(9)**	529	0.03
4	Glc*N*Ac[1,3″]Le^x^ **(10)**	18637	2.1

*^a^* Concentration of inhibitor required for 50% inhibition using DimLe^x^-BSA conjugate **1** as immobilized antigen. *^b^* Values determined from the expression Δ(ΔG) = RT ln([I_1_]/[I_2_] where [I_2_] is the IC_50_ measured for the reference inhibitor DimLe^x^ **6**, and [I_1_] is the IC_50_ measured for each fragment with R = 1.98 cal K^−1^and T = 296 K.

**Table 2:** Inhibition data for mAb SH2, SH1, and 1G5F6 with Le^x^ analogues **7**, **15**–**22**.

		SH2		SH1 *^d^*	1G5F6 *^e^*
Entry	Inhibitor	IC_50_ *^a^* (μM)	∆(∆G) *^b^* (kcal·mol^−1^)	∆(∆G) *^d^* (kcal·mol^−1^)	∆(∆G) *^e^* (kcal·mol^−1^)
1	Le^x^ **(7)**	47	0	0	0
2	LacLe^x^ **(15)**	>>1800	---*^c^*	0.2	2.5
3	GlcLe^x^ **(16)**	>>1800	---*^c^*	---*^c^*	2.7
4	RhaLe^x^ **(17)**	827	1.7	1.1	1.6
5	RhaLac **(18)**	>>1800	---*^c^*	1.5	3.2
6	4″-MeOLe^x^ **(19)**	148	0.7	---*^c^*	---*^c^*
7	4″-HLe^x^ **(20)**	74	0.3	1.3	0.3
8	4″-ClLe^x^ **(21)**	19	-0.5	1.6	0.3
9	4″-FLe^x^ **(22)**	17	-0.6	2.1	−0.1

*^a^* Concentration of inhibitor required for 50% inhibition using DimLe^x^-BSA conjugate **1** as immobilized antigen. *^b^* Values determined from the expression Δ(ΔG) = RT ln([I_1_]/[I_2_] where [I_2_] is the IC_50_ measured for the reference inhibitor Le^x^ **7** (shaded in yellow), and [I_1_] is the IC_50_ measured for each analogue **15–22** with R = 1.98 cal K^−1^and T = 296 K. *^c^* No inhibition. *^d^* Inhibition data published [36,37] for SH1 using Le^x^-BSA conjugate **4** as immobilized antigen and Le^x^ trisaccharide **7** as reference. *^e^* Inhibition data published [35] for 1G5F6 using DimLe^x^-BSA conjugate **1** as immobilized antigen and Le^x^ trisaccharide **7** as reference.

## Supplementary Materials

The following are available online at https://www.mdpi.com/2076-393X/8/3/538/s1, Figure S1: Titration curves for SH2 ascites with various coating concentrations of conjugate (DimLe^x^)_16_-BSA **1**.
