# Supplementary material for: Recognition of Dimeric Lewis X by Anti-Dimeric Lex Antibody SH2
Source: Vaccines (Basel). 2020 Sep 17;8(3):538. doi: 10.3390/vaccines8030538 (PMC7563222; doi:10.3390/vaccines8030538)

Titration curves for SH2 ascites with various coating concentrations of conjugate $(DimLe^{x})_{16}$-BSA **1**

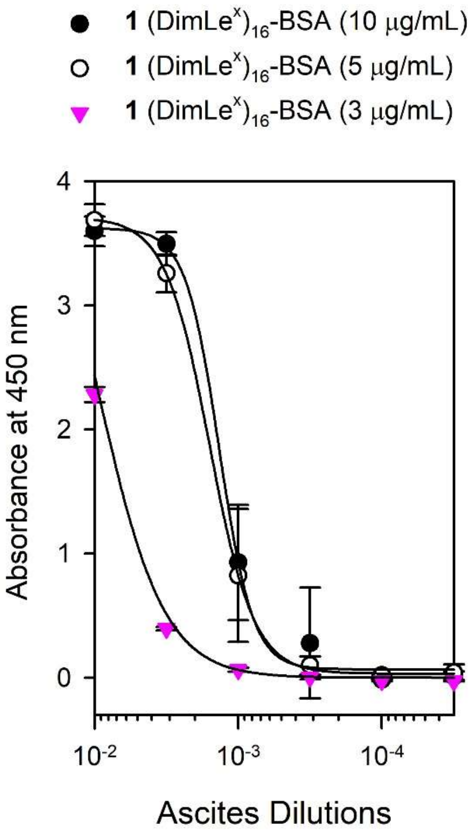

Supplement: Supplementary file 1 [file vaccines-08-00538-s001.pdf]
